# Expression of Signaling Components in Embryonic Eyelid Epithelium

**DOI:** 10.1371/journal.pone.0087038

**Published:** 2014-02-03

**Authors:** Qinghang Meng, Chang Jin, Yinglei Chen, Jing Chen, Mario Medvedovic, Ying Xia

**Affiliations:** Department of Environmental Health, College of Medicine, University of Cincinnati, Cincinnati, Ohio, United States of America; Wayne State University, United States of America

## Abstract

Closure of an epithelium opening is a critical morphogenetic event for development. An excellent example for this process is the transient closure of embryonic eyelid. Eyelid closure requires shape change and migration of epithelial cells at the tip of the developing eyelids, and is dictated by numerous signaling pathways. Here we evaluated gene expression in epithelial cells isolated from the tip (leading edge, LE) and inner surface epithelium (IE) of the eyelid from E15.5 mouse fetuses by laser capture microdissection (LCM). We showed that the LE and IE cells are different at E15.5, such that IE had higher expression of muscle specific genes, while LE acquired epithelium identities. Despite their distinct destinies, these cells were overall similar in expression of signaling components for the “eyelid closure pathways”. However, while the LE cells had more abundant expression of *Fgfr2*, *Erbb2*, *Shh*, *Ptch1* and *2*, *Smo* and *Gli2*, and *Jag1* and *Notch1*, the IE cells had more abundant expression of *Bmp5* and *Bmpr1a*. In addition, the LE cells had more abundant expression of *adenomatosis polyposis coli down-regulated 1* (*Apcdd1*), but the IE cells had high expression of *Dkk2*. Our results suggest that the functionally distinct LE and IE cells have also differential expression of signaling molecules that may contribute to the cell-specific responses to morphogenetic signals. The expression pattern suggests that the EGF, Shh and NOTCH pathways are preferentially active in LE cells, the BMP pathways are effective in IE cells, and the Wnt pathway may be repressed in LE and IE cells via different mechanisms.

## Introduction

Formation of the eyelid is one of the last major morphogenetic events in mammalian prenatal development. Though for the most part data are scarce in humans, histological analyses of available embryos/fetuses have shown that eyelid development proceeds through four distinct phases, namely, lid formation, growth, fusion and re-opening [1], [2]. In mice, eyelid development follows similar steps but has been characterized in greater detail. Mouse eyelid formation begins at around embryonic day 11.5 (E11.5). At this time, the surface ectoderm adjacent to the developing cornea folds to form the lid buds, which are a simple structure consisting of loose periocular mesenchyme (POM) covered by undifferentiated ectoderm [3]–[6]. The eyelid buds grow from E12 onward, and they extend across the ocular surface, undergoing proliferation and differentiation. The eyelid at this stage is covered by epidermis, overlaid by periderm at the anterior surface and conjunctiva at the posterior surface. The epithelial margins of the superior and inferior lid fuse between E15 - E16. Lid fusion begins when the periderm cells become rounded and piled up at the leading edges of the eyelids, and then stream out across the corneal surface. The eyelids meet at the inner and outer canthi and temporarily fuse across the cornea [3], [4]. Once contact is established between the apposed eyelids, the cells at the fusion junction flatten and form a strip along the fusion line, and they slough off with the rest of the periderm [4], [7], [8]. Mouse eyelid remains closed between E16.5 and postnatal day 12–14. Cells at the eyelid fusion junction undergo desquamation and/or apoptosis, resulting in separation of the upper and lower eyelids at around postnatal day 14 [4], [9].

Much is known about the molecular factors involved in eyelid formation and fusion. This is because, although mice are normally born with a closed eyelid, a large number of genetic mutant strains display a distinct “eye open at birth” (EOB) phenotype. The Mouse Genome Informatics (MGI) (http://www.informatics.jax.org/) has a collection of >138 genotypes associated with the phenotype; the number is likely to increase with complete or partial knockout of new genes.

The majority of the EOB phenotype is caused by failure of eyelid fusion at E15–E16. One of the most significant findings made by the analysis of EOB mice is that multiple signaling pathways are involved in the regulation of eyelid closure. Some pathways, such as RA-RXR/RAR and PITX2-DKK2, and the FOXL and OAR2 transcription factors, seem to operate in the periocular mesenchyme [10]–[12]; others, such as the FGF10-FGFR and BMP-BMPR pathways, act through crosstalk between mesenchyme and epithelium [6], [13]. Furthermore, a number of pathways, including MAP3K1-JNK, EGFR, ROCK and PCP, are specifically effective in the eyelid epithelial cells [14]–[35]. There is also evidence for signal compartmentalization and spatial segregation, so that the signaling pathways are activated in distinct cell population in the developing eyelids [21], [36].

Though the outline of the pathways is more or less drawn, the role that the actual players involved in signal transduction has not been fully understood. Genetic knockout studies in mice have helped to elucidate the roles of some of the signaling molecules. Using this approach, it is shown that multiple EGFR ligands act additively to regulate eyelid morphogenesis. Thus, whereas the *Hb-egf*-null and *Tgfα*-null mice display occasionally “open-eye” phenotype, the compound mutants, i.e. *Hb-egf(−/−)Tgfα(+/−)* and *Hb-egf(+/−)Tgfα(−/−)*, have a slightly increased penetrance, and the double homozygous null mice have a drastically increased penetrance of the phenotype. Furthermore, the triple null mice, lacking three of the EGFR ligand genes, *Egf*, *Areg* and *Tgfα*, exhibit a severe “eye-open” phenotype [37]. Similarly, by generating a series of genetic mutant strains, Huang, *et. al*. have shown the BMP signaling is specifically involved in eyelid closure. Mice lacking components of the TGFβ pathways have normal eyelid development, but those with impaired BMP signaling display an ‘eyelid open at birth’ phenotype [13].

The most remarkable feature of lid closure is the shape change and migration underwent by the epithelial cells at the “tip” of the eyelid. This is accompanied by activation of specific morphogenetic pathways. It is possible that the tip cells have unique surrounding tissues, i.e., microenvironments, which produce morphogens for specific activation of signaling pathways. Alternatively, the tip cells may have unique gene expression thereby acquiring new signaling and morphogenetic properties. Gene expression is a crucial facet of its function, and many genes essential for eyelid closure, such as *Tgfα*, *Hb-egf*, *Activinβb* and *Map3k1*, are indeed up-regulated in the developing eyelid epithelium [6], [20], [38], [39].

In the present work, we applied a global approach to compare gene expression profiles in epithelial cells isolated from the tip (leading edge, LE) and the inner surface (inner epithelium, IE) of the embryonic eyelid. We evaluated the relative abundance in expression of genes whose products might constitute the major “eyelid closure pathways”. Results may help to understand how signals are distinctly regulated in the LE cells and provide guidance for selecting “genes of interest” for expression and knockout studies.

## Materials and Methods

### Experimental animals

C57BL/6 fetuses were collected at E15.5. Euthanasia of the E15.5 fetuses was done by decapitation with surgical scissors, and genotypes were determined by PCR. Experiments conducted with these animals were carried out in strict accordance with the recommendations in the Guide for the Care and Use of Laboratory Animals of the National Institutes of Health. The protocol was approved by the Committee on the Ethics of Animal Experiments of the University of Cincinnati (Protocol no. 06-04-19-01).

### Tissue and cell preparation, RNA and cDNA generation and microarray

This process was done as previously described [40]. Briefly, the heads of E15.5 fetuses were embedded in Tissue-Tek OCT medium (Sakura Finetek USA) and stored in −80°C. Eight µm coronal sections were mounted on plain uncoated glass slides, dehydrated and stained with HistoGene LCM frozen section staining kit, and were used for LCM following the manufacturer's protocol (Molecular Devices). Cells from 4 sections were collected on one LCM cap and lysed for RNA harvesting. The lysates from each fetus were pooled and processed as one biological sample. It was estimated that 10 ng and 15 ng total RNA were obtained from LE and IE eyelid epithelium, respectively, per fetus.

RNA was analyzed by Agilent 2100 Bioanalyzer (Agilent, Santa Clara, CA) and samples with RNA Integrity Number (RIN) >5.5 were processed for cDNA amplification. cDNA amplification and biotinylation was done using Ovation Pico WTA System (NuGEN, San Carlos, CA) following the manufacturer's instructions. Specifically, RNA (10 ng) was processed into first strand cDNA, a DNA/RNA heteroduplex, and thereafter a linear isothermal amplified cDNA. The amplified cDNA was purified with a PCR Purification Kit (QIAGEN, Valencia, CA).

The cDNAs from each fetus were considered one biological sample and 3 samples were used for triplicate hybridization on the Affymetrix GeneChip Mouse Gene 1.0 ST array (P/N 901168, Affymetrix, Santa Clara, CA). The arrays were hybridized with 15 µg of fragmented aRNA. The hybridization, staining, and washing are carried out using the Affymetrix GeneChip Hybridization Wash and Stain Kit (P/N 900720) following the manufacturer's protocols. The arrays were hybridized for 16 hr at 45°C using Affymetrix Hybridization Oven 640 (P/N 800139). FS450-0001 protocol was used for staining and washing the GeneChips using the Affymetrix Fluidics Station 450 (P/N 00-0079). The GeneChips were scanned with Affymetrix GeneChip Scanner 3000 7G Plus using Affymetrix GeneChip Command Console 3.2.3.1515 software and Affymetrix preset settings.

### Quantitative RT-PCR

Quantitative PCR was performed using an MX3000p thermal cycler system and SYBR Green QPCR Master Mix (Stratagene), using conditions optimized for each target gene primers with efficiency greater than 85%, cycles less than 29 and sample locations on the plates been randomized. The PCR products were subjected to melting curve analysis and the relative cycle differences in qRT-PCR were determined using ΔCt, as described [41]. The ΔCt value for each sample was determined using the cycle threshold (Ct) value of the specific gene normalized to that of *Gapdh*. The fold change was calculated based on the ratio between LE versus IE (control) samples, designated as 1. Data are based on triplicate reactions of at least 3 biological samples.

### Statistical and bioinformatics analyses

Array data (GEO repository, accession no. GSE39240) were analyzed at gene level using statistical software R and the limma package of Bioconductor [42] with custom CDF downloaded from BrainArray [43]. Data pre-processing, including background correction and normalization, was performed using RMA. Array quality was assessed using the Array Quality Metrics package of Bioconductor [44]. Statistical significance of differential gene expression between LE and IE samples were established based on empirical Bayes linear model as implemented in limma package [42].

Functional enrichment analysis of differentially expressed genes was performed using the logistic regression based LRpath methodology [45] as implemented in the R package CLEAN [46]. The gene list used in the functional enrichment analysis came from genes associated with Gene Ontology terms [47]. The statistical significance of gene list enrichment was determined based on the False Discovery Rate (fdr) cut-off of 0.1. The statistical significance of deviations of average gene expression levels for genes within the same group were established by calculating gene specific z-statistics and comparing it to the standard Normal distribution. The z-statistic was calculated by subtracting the average of expression levels of all genes in the group from the expression level of the gene and dividing the difference by the standard deviation of the expression levels within the group.

## Results and Discussion

### Gene expression profiles in the developing eyelid epithelium

To identify the molecular signatures of eyelid closure, we collected mouse fetuses at E15.5, a developmental stage immediately before the eyelid beginning to close. We used laser capture microdissection (LCM) to isolate epithelial cells from the leading edge (LE) and inner surface epithelium (IE). The samples were used for expression array and gene expression signatures were analyzed as described [40].

To determine whether the LE and IE cells were different at E15.5, we analyzed the expression data by Gene Ontology (GO). The LE cells were enriched for genes involved in epidermis development, transcription factor activity, pattern specification and odontogenesis. By contrast, the IE cells were enriched for genes for muscle development, RNA splicing, microtubule organization and centrosomes (Table 1). The GO signatures suggest that the E15.5 LE and IE cells have already departed to distinct paths from their common origin - the ocular surface ectoderm.

**Table 1:** Gene Functions in LE and IE Cells.

categoryID	description	nGenes	zScore	pValue	FDR
**Up-regulated in LE cells**					
GO:0008544	epidermis development	202	9.513937667	9.18E-22	4.91E-18
GO:0001071	nucleic acid binding transcription factor activity	725	7.756885322	4.35E-15	7.76E-12
GO:0007389	pattern specification process	389	6.372488177	9.30E-11	6.78E-08
GO:0042475	odontogenesis of dentin-containing tooth	56	6.359209662	1.01E-10	6.78E-08
**Up-regulated in IE cells**					
GO:0005865	striated muscle thin filament	15	9.710632193	1.35823E-22	7.26656E-19
GO:0008380	RNA splicing	222	7.022839172	1.08702E-12	2.90778E-09
GO:0005815	microtubule organizing center	353	5.980688777	1.11098E-09	8.49106E-07
GO:0005813	centrosome	334	5.947685008	1.35981E-09	9.09371E-07

### Expression of signaling molecules in the FGF and EGF pathways

To evaluate whether the LE and IE cells had differential expression of signaling molecules, we examined genes involved in the FGF and EGF pathways, known to be involved in eyelid closure. The fibroblast growth factor (FGF) family has 22 ligands and four membrane-bound receptors, FGFR 1-4, with different ligand binding affinities [48], [49]. In LE and IE cells, the *Fgfr2* was the most abundantly expressed receptor gene, and *Fgf9* was the highly expressed ligand gene (Table 2). Between LE and IE, there was no major difference in the expression of genes belonging to the families of FGF ligands and receptors, except for *Fgfr2* (Table 2). The level of *Fgfr2* was 1.8-fold higher in LE cells, suggesting that the LE cells might be more responsive to FGF signals than the IE cells.

**Table 2:** Expression of Genes in the FGF pathways

		LE		IE		LE/IE	
symbol	name	ave.int	p-value	ave.int	p-value	fold	p.value
The FGF family							
***Ligands***							
*Fgf9*	fibroblast growth factor 9	**391.7362245**	**0.002337928**	**390.6433611**	**0.019267398**	1.002797599	0.240347549
*Fgf8*	fibroblast growth factor 8	166.5464506	0.155681338	168.861405	0.535466303	-1.013899752	0.2104111
*Fgf22*	fibroblast growth factor 22	126.5957788	0.368634212	159.4079042	0.616024893	-1.259188147	0.291215529
*Fgf17*	fibroblast growth factor 17	125.059223	0.381111278	120.5839736	0.943298623	1.037113136	0.142005947
*Fgf15*	fibroblast growth factor 15	106.552639	0.567451803	196.9766114	0.349464154	-1.848631936	0.423612008
*Fgf18*	fibroblast growth factor 18	100.7913239	0.641017588	210.65336	0.283116505	-2.08999497	0.519448518
*Fgf13*	fibroblast growth factor 13	89.66438499	0.807082058	189.7867092	0.390149852	-2.11663426	0.303068764
*Fgf23*	fibroblast growth factor 23	87.28123282	0.846919936	129.6743787	0.937844546	-1.48570746	0.083917121
*Fgf12*	fibroblast growth factor 12	85.02659868	0.886003145	165.5686431	0.562397439	-1.947257043	0.183738989
*Fgf14*	fibroblast growth factor 14	81.91780637	0.942085876	151.3383997	0.692901632	-1.847442045	0.860187995
*Fgf4*	fibroblast growth factor 4	77.78685355	0.979586236	99.0757551	0.635380846	-1.273682513	0.229425806
*Fgf2*	fibroblast growth factor 2	70.66744472	0.835369348	217.5071721	0.254690818	-3.077897793	0.13672465
*Fgf3*	fibroblast growth factor 3	68.74815066	0.794789854	90.72130986	0.512539018	-1.319618186	0.335560338
*Fgf11*	fibroblast growth factor 11	68.56522928	0.790890829	75.35099222	0.300332225	-1.09896799	0.170136214
*Fgf20*	fibroblast growth factor 20	66.76075364	0.752163879	108.3953639	0.772018719	-1.623639009	0.832224495
*Fgf5*	fibroblast growth factor 5	56.66072417	0.53052365	83.88846438	0.414768503	-1.480539926	0.545076451
*Fgf6*	fibroblast growth factor 6	56.09740044	0.518165165	61.21500656	0.14375436	-1.091227153	0.004820016
*Fgf1*	fibroblast growth factor 1	50.52878074	0.39830177	66.54665181	0.196832397	-1.3170049	0.871058322
*Fgf16*	fibroblast growth factor 16	46.44946676	0.315151851	83.9707348	0.415920032	-1.807786842	0.213019604
*Fgf21*	fibroblast growth factor 21	43.45787888	0.258114299	80.28778119	0.365183695	-1.847485042	0.47884619
*Fgf7*	fibroblast growth factor 7	40.01974398	0.197978384	177.3818719	0.47111134	-4.432358986	0.137301471
*Fgf10*	fibroblast growth factor 10	39.45348786	0.188715937	62.20935837	0.153034151	-1.576777156	NA
***Receptors***							
*Fgfr2*	fibroblast growth factor receptor 2	813.1860577	0.193619621	451.5885556	0.235326753	**1.800723352**	**0.01626331**
*Fgfr1*	fibroblast growth factor receptor 1	300.4716334	0.813353765	255.3859332	0.794570092	1.176539482	0.179540765
*Fgfr3*	fibroblast growth factor receptor 3	140.6777924	0.565431926	188.2482391	0.813949118	-1.338151785	1
*Fgfr4*	fibroblast growth factor receptor 4	97.98335351	0.336423871	103.2283889	0.225590878	-1.053529862	0.145817534

Previously, we have shown that FGF9 expression was decreased in LE cells of *Map3k1* knockout fetuses corresponding to failure of eyelid closure [40]. FGF9 could act in an autocrine fashion to induce epithelial branching, or it could send signals to the mesenchyme to induce PITX2 and FGF10. FGF10 in turn could trans-activate FGFR in epithelial cells and stimulate epithelial budding [50], [51]. Genetic studies show that FGF10 is crucial for eyelid closure, but FGF9, though required for sex determination and reproductive system development, lung embryogenesis, and inner ear morphogenesis, is dispensable for eyelid development [52]–[54]. Since FGF10 was almost undetectable in LE and IE cells, it is possible that this ligand is produced by the underlying mesenchymal cells, responsible for activation of FGFR2 in the eyelid epithelium [5], [6].

The epidermal growth factor (EGF) pathway operates in an autocrine fashion, such that ligands produced by the epithelial cells can activate receptors on the same or nearby cells [6], [38], [55], [56]. The mammalian system has nine ligands, which are first expressed as transmembrane proteins comprising a signal sequence, a transmembrane domain and the EGF domain(s). The ligands are then activated by ectodomain shedding that releases the EGF domain from the membrane-bound precursors. This is carried out by members of disintegrin and metalloproteases (ADAMS) family of type I transmembrane Zn-dependent proteases. There are four EGF receptor tyrosine kinases, including EGFR/ERBB1, ERBB2, ERBB3 and ERBB4 [57]. Activation of the receptors is also facilitated by members of the leucine-rich repeat containing G-protein coupled receptor (LGR) and G protein-coupled receptor (GPCR) families.

In LE and IE cells, the *Egfr* and *Erbb2*, and several genes in the GPCR families, such as *Lgr4*, *Gpr125*, *Gpr20, Gpr180*, *Gpr89* and *Gpr3*, were abundantly expressed (Tables 3 and 4). Expression of *Adams10* was also abundant (Table 5). Expression of *Gpr56* was relatively abundant in LE cells, whereas expression of *Adam 17*, *Lgr4*, *Gpr107* and *Gpr137b-ps* was more abundant in IE cells. Compared to the IE cells, the LE cells had significantly higher expression of *Erbb2* (1.8-fold) and *Gpr56* (1.3-fold), but less expression of *Adamts1* (-2.6-fold).

**Table 3:** Expression of Genes in the EGF pathways

		LE		IE		LE/IE	
symbol	name	ave.int	p-val	ave.int	p-val	fold	p.val
The EGF family							
***Ligands***							
*Areg*	amphiregulin	155.3082961	0.133546244	196.8743744	0.233004491	-1.267635917	0.478968664
*Hbegf*	heparin-binding EGF-like growth factor	128.9845997	0.297282478	167.4743744	0.384089575	-1.298405971	0.478845479
*Tgfa*	transforming growth factor alpha	105.2105058	0.589279991	153.7290675	0.484086582	-1.461157005	0.202676617
*Nrg1*	neuregulin 1	98.00624272	0.715140496	155.8413258	0.467264689	-1.590116318	0.685383852
*Btc*	betacellulin, epidermal growth factor family member	87.55213556	0.930827589	117.8289953	0.865162398	-1.345815205	0.715209746
*Nrg2*	neuregulin 2	83.82453012	0.983653113	79.467853	0.538534554	1.054823139	NA
*Nrg3*	neuregulin 3	58.13060811	0.355939687	103.0507744	0.922549334	-1.772745509	0.478847044
*Ereg*	epiregulin	52.18751712	0.234405883	55.55150234	0.184028108	-1.064459576	0.565380436
*Egf*	epidermal growth factor	47.88076511	0.161070096	48.01846247	0.105485701	-1.002875839	0.478845479
***Receptors***							
*Erbb2*	v-erb-b2 erythroblastic leukemia viral oncogene homolog 2	400.0763611	0.357543272	224.2186429	0.920362594	**1.784313543**	**0.018738394**
*Egfr*	epidermal growth factor receptor	268.515161	0.647969842	410.0439401	0.18271137	-1.527079285	0.478850402
*Erbb3*	v-erb-b2 erythroblastic leukemia viral oncogene homolog 3	184.0990756	0.985729198	174.7380987	0.682470129	1.05357147	0.273357054
*Erbb4*	v-erb-a erythroblastic leukemia viral oncogene homolog 4	54.61474781	0.163158368	129.3400888	0.30614936	-2.368226422	0.472018438

**Table 4:** Expression of genes in the *Lgr* and *Gpr* families

		LE		IE		LE/IE	
symbol	name	ave.int	p-val	ave.int	p-val	fold	p.val
***Lgr***							
*Lgr4*	leucine-rich repeat-containing G protein-coupled receptor 4	157.3388508	0.404500028	383.3124767	0.25234085	-2.43622268	0.287853304
*Lgr6*	leucine-rich repeat-containing G protein-coupled receptor 6	116.419403	0.783191878	103.8732732	0.481567664	1.120783041	0.129157861
*Lgr5*	leucine rich repeat containing G protein coupled receptor 5	55.18917435	0.267528025	125.0686009	0.659286312	-2.266179959	0.596789619
***Gpr***							
*Gpr125*	G protein-coupled receptor 125	220.1038045	0.021084389	289.8375491	0.026009754	-1.316822078	0.500824711
*Gpr56*	G protein-coupled receptor 56	218.8178318	0.021853992	171.4743744	0.303240853	1.2760964	0.01999301
*Gpr20*	G protein-coupled receptor 20	211.4585922	0.026852976	316.8945328	0.01511878	-1.498612705	0.839414104
*Gpr35*	G protein-coupled receptor 35	180.0479953	0.065600296	211.299815	0.132169905	-1.173574938	0.746554847
*Gpr180*	G protein-coupled receptor 180	175.9631725	0.073778453	225.2654962	0.098636269	-1.280185467	0.478842536
*Gpr89*	G protein-coupled receptor 89	175.0870638	0.075663315	259.1367522	0.04874379	-1.480045108	0.860737542
*Gpr3*	G-protein coupled receptor 3	166.2545002	0.097617959	256.2424001	0.051748411	-1.541265949	0.33371539
*Gpr27*	G protein-coupled receptor 27	164.4406301	0.102869023	147.2047335	0.495484149	1.117087924	0.000579647
*Gpr107*	G protein-coupled receptor 107	162.419388	0.109055871	229.0743744	0.09108073	-1.410388114	0.137248811
*Gpr108*	G protein-coupled receptor 108	145.2163784	0.17923789	191.0189953	0.202107027	-1.315409442	0.911106857
*Gpr137*	G protein-coupled receptor 137	127.2392185	0.299750484	136.5343716	0.61000012	-1.073052579	0.203778073
*Gpr135*	G protein-coupled receptor 135	124.301855	0.325673932	117.9937349	0.859217628	1.053461483	0.370495905
*Gpr119*	G-protein coupled receptor 119	123.7604085	0.330676321	130.9329503	0.678452003	-1.057955059	0.157171461
*Gpr75*	G protein-coupled receptor 75	123.6542976	0.331665052	135.7166762	0.619626637	-1.097549207	0.216351769
*Gpr44*	G protein-coupled receptor 44	123.3665465	0.334360194	130.317495	0.686333622	-1.056343868	0.496977287
*Gpr124*	G protein-coupled receptor 124	115.0903184	0.42110489	149.9910593	0.468845533	-1.303246541	0.478842349
*Gpr39*	G protein-coupled receptor 39	97.00347273	0.682938697	105.2282022	0.933334644	-1.08478799	0.33371539
*Gpr123*	G protein-coupled receptor 123	96.88645603	0.684992273	125.8634937	0.745514901	-1.29908244	0.247272771
*Gpr85*	G protein-coupled receptor 85	96.61297529	0.689810486	139.7885876	0.572910687	-1.446892481	0.674432534
*Gpr30*	G protein-coupled receptor 30	96.58976307	0.690220652	107.3732646	0.969969091	-1.111642281	0.186297098
*Gpr153*	G protein-coupled receptor 153	94.94188296	0.719823958	98.13033433	0.808122405	-1.033583191	0.312765421
*Gpr137b-ps*	G protein-coupled receptor 137B, pseudogene	94.78743922	0.722647465	282.2968287	0.030313391	-2.978209255	0.680474406
*Gpr81*	G protein-coupled receptor 81	92.51193082	0.765219363	123.7269258	0.775238854	-1.337415884	0.469995365
*Gpr4*	G protein-coupled receptor 4	92.03372555	0.774396617	103.5068225	0.903492859	-1.124661877	0.85686891
*Gpr179*	G protein-coupled receptor 179	91.13654808	0.791829182	100.0755366	0.842964993	-1.098083466	0.327688347
*Gpr97*	G protein-coupled receptor 97	90.49460245	0.804473656	96.21870568	0.773579498	-1.063253532	0.341054528
*Gpr172b*	G protein-coupled receptor 172B	88.72249257	0.8401129	97.276255	0.792722632	-1.096410303	0.037918301
*Gpr171*	G protein-coupled receptor 171	85.5606136	0.906315064	191.2743744	0.201031884	-2.235542341	0.288166398
*Gpr25*	G protein-coupled receptor 25	84.40014173	0.931423514	98.44886788	0.813850833	-1.166453822	0.065281383
*Gpr6*	G protein-coupled receptor 6	82.59264406	0.971357511	74.27593798	0.380077813	1.111970395	0.815470951
*Gpr137b*	G protein-coupled receptor 137B	81.25127761	0.998384708	107.3979928	0.970387644	-1.321800665	0.713122571
*Gpr114*	G protein-coupled receptor 114	80.90069545	0.990392729	82.19300262	0.517720661	-1.015973993	0.496210735
*Gpr17*	G protein-coupled receptor 17	80.64630589	0.984572436	96.65240053	0.781439379	-1.198472757	0.48758246
*Gpr173*	G-protein coupled receptor 173	80.57298468	0.982891617	102.3705866	0.883595209	-1.270532387	0.865882429
*Gpr83*	G protein-coupled receptor 83	77.34451159	0.907515055	118.4744165	0.851944372	-1.531775352	0.664800049
*Gpr183*	G protein-coupled receptor 183	75.90513232	0.873125838	136.6792593	0.608307331	-1.800658995	0.977344711
*Gpr133*	G protein-coupled receptor 133	73.77656211	0.821530114	92.57027338	0.707052638	-1.254738236	0.310248937
*Gpr126*	G protein-coupled receptor 126	72.44315307	0.788832074	232.0775545	0.085538588	-3.203581631	0.138923358
*Gpr18*	G protein-coupled receptor 18	72.21733384	0.783270351	182.9476886	0.239134407	-2.533293309	0.501943232
*Gpr84*	G protein-coupled receptor 84	72.20812693	0.783043458	86.08696737	0.588271302	-1.192206072	0.224588613
*Gpr37l1*	G protein-coupled receptor 37-like 1	72.13222905	0.781172645	98.78720469	0.819925994	-1.369529349	0.431585749
*Gpr144*	G protein-coupled receptor 144	72.06972089	0.779631349	79.31147279	0.466480372	-1.100482586	0.132102738
*Gpr157*	G protein-coupled receptor 157	71.40005822	0.763090782	96.67623462	0.781870956	-1.354007784	0.358178624
*Gpr156*	G protein-coupled receptor 156	70.40086729	0.738326035	68.5804486	0.289316439	1.026544281	0.478847201
*Gpr146*	G protein-coupled receptor 146	69.62364684	0.719006489	53.54305799	0.104400524	1.300330042	0.13090816
*Gpr160*	G protein-coupled receptor 160	69.39114229	0.713219524	74.72537227	0.387587547	-1.076871915	0.337863882
*Gpr77*	G protein-coupled receptor 77	69.38658457	0.713106053	64.65720239	0.232512432	1.073145481	0.246294322
*Gpr68*	G protein-coupled receptor 68	65.98359522	0.628259898	89.82589824	0.656736889	-1.361336828	0.836126668
*Gpr162*	G protein-coupled receptor 162	63.89859714	0.576464248	71.74780097	0.338720298	-1.122838437	0.753129884
*Gpr132*	G protein-coupled receptor 132	61.01745433	0.505773427	65.42941991	0.243272256	-1.072306615	0.770153611
*Gpr111*	G protein-coupled receptor 111	60.35405182	0.489718441	54.49499311	0.113259572	1.107515542	0.379862366
*Gpr62*	G protein-coupled receptor 62	58.81170703	0.45281891	71.99871533	0.342753671	-1.224224206	0.438288689
*Gpr26*	G protein-coupled receptor 26	58.61960404	0.448269774	80.90301094	0.494654237	-1.380135746	0.470158493
*Gpr15*	G protein-coupled receptor 15	57.40978461	0.419890293	57.81324406	0.147347983	-1.007027712	0.153990594
*Gpr161*	G protein-coupled receptor 161	55.91072381	0.385447822	107.498657	0.972090592	-1.922684052	0.512145141
*Gpr45*	G protein-coupled receptor 45	55.40839734	0.374105939	53.82411607	0.106973001	1.029434413	0.140431857
*Gpr151*	G protein-coupled receptor 151	54.78053147	0.360082493	85.45866943	0.576815937	-1.560018991	1
*Gpr65*	G-protein coupled receptor 65	54.05701451	0.344145124	146.8162858	0.499301801	-2.715952538	0.158633441
*Gpr182*	G protein-coupled receptor 182	53.23426477	0.326329103	85.11797113	0.57061356	-1.598932032	0.567583183
*Gpr139*	G protein-coupled receptor 139	53.02682408	0.321891129	92.38956386	0.703743558	-1.742317506	0.258601234
*Gpr21*	G protein-coupled receptor 21	52.0328213	0.300942497	72.73913052	0.354750161	-1.397947078	0.741406994
*Gpr158*	G protein-coupled receptor 158	51.92947168	0.29879543	63.41336159	0.215644175	-1.221143977	0.637090301
*Gpr63*	G protein-coupled receptor 63	50.9959338	0.279678293	97.86797672	0.803397981	-1.919132947	0.946152571
*Gpr155*	G protein-coupled receptor 155	49.97110494	0.259290745	92.12694599	0.698933085	-1.843604341	0.324933584
*Gpr176*	G protein-coupled receptor 176	48.96242303	0.239872194	53.89486012	0.107626211	-1.100739236	0.212932186
*Gpr19*	G protein-coupled receptor 19	48.77902167	0.236413235	88.48209068	0.632092019	-1.813937378	0.478842035
*Gpr116*	G protein-coupled receptor 116	47.64519495	0.215538201	95.65763979	0.763393274	-2.007708015	0.237971027
*Gpr37*	G protein-coupled receptor 37	46.63567635	0.197714832	78.6480309	0.45484197	-1.686434873	0.33350725
*Gpr12*	G-protein coupled receptor 12	43.87164829	0.152825917	89.07378877	0.642941053	-2.030326925	0.25456221
*Gpr61*	G protein-coupled receptor 61	38.39824197	0.082156744	90.09705371	0.661711292	-2.346384863	0.725656569
*Gpr137c*	G protein-coupled receptor 137C	38.28835404	0.080994315	89.45583486	0.649948364	-2.33637191	NA

**Table 5:** Expression of genes in the Adams family

		LE		IE		LE/IE	
symbol	name	ave.int	p-val	ave.int	p-val	fold	p.val
***Adams***							
*Adam10*	a disintegrin and metallopeptidase domain 10	**361.9310254**	**0.002605289**	**572.1144261**	**0.002197908**	-1.580727779	0.550173668
*Adamtsl4*	ADAMTS-like 4	186.7402441	0.118746409	183.2381962	0.566007252	1.019111997	0.211012267
*Adam17*	a disintegrin and metallopeptidase domain 17	179.240216	0.14151102	327.0666618	0.065754322	-1.824739275	0.43979387
*Adam15*	a disintegrin and metallopeptidase domain 15	176.91194	0.149437176	236.3028883	0.258585267	-1.335709101	0.86373439
*Adamts17*	a disintegrin-like and metallopeptidase with thrombospondin type 1, 17	166.285723	0.191649211	155.1157197	0.83380408	1.072010776	0.17838096
*Adam33*	a disintegrin and metallopeptidase domain 33	143.2533418	0.327655809	187.3018386	0.534022951	-1.307486696	0.607122322
*Adamts10*	a disintegrin-like and metallopeptidase with thrombospondin type 1, 10	135.1273739	0.394870436	188.5363387	0.524624481	-1.39524904	0.323396275
*Adam1a*	a disintegrin and metallopeptidase domain 1a	125.7803888	0.487891176	142.3710583	0.982078417	-1.131901878	0.302441913
*Adamtsl5*	ADAMTS-like 5	118.8409891	0.569188872	156.684171	0.816684342	-1.318435434	0.219403185
*Adamts2*	a disintegrin-like and metallopeptidase with thrombospondin type 1, 2	114.1361944	0.630738755	178.4675506	0.605650924	-1.563636772	0.981455083
*Adamts1*	a disintegrin-like and metallopeptidase with thrombospondin type 1, 1	109.4934689	0.696800634	288.2065321	0.117893987	-2.632180121	0.032615535
*Adamts8*	a disintegrin-like and metallopeptidase with thrombospondin type 1, 8	108.1270441	0.717269221	137.1852326	0.953251485	-1.268741171	0.670359886
*Adamts7*	a disintegrin-like and metallopeptidase with thrombospondin type 1, 7	104.7353254	0.770104259	126.3711396	0.811832594	-1.206576091	0.419966026
*Adamts12*	a disintegrin-like and metallopeptidase with thrombospondin type 1, 12	103.266259	0.793884786	253.7738818	0.198575014	-2.457471436	0.15311868
*Adam4*	a disintegrin and metallopeptidase domain 4	101.4912963	0.823334302	103.9723183	0.506418509	-1.024445663	0.359728774
*Adam11*	a disintegrin and metallopeptidase domain 11	96.48278062	0.910581493	93.32233476	0.367807843	1.033865911	0.16144701
*Adam9*	a disintegrin and metallopeptidase domain 9	92.75365702	0.979347597	188.8591255	0.522191173	-2.036136705	0.321859941
*Adamtsl2*	ADAMTS-like 2	90.38008471	0.975311941	104.9617327	0.519752615	-1.161336959	0.15311868
*Adamtsl1*	ADAMTS-like 1	89.39194852	0.956099857	117.7630678	0.694883169	-1.317378911	0.689678828
*Adam19*	a disintegrin and metallopeptidase domain 19	89.37741331	0.955815834	139.0754455	0.977089116	-1.556046884	0.632565024
*Adamts16*	a disintegrin-like and metallopeptidase with thrombospondin type 1, 16	89.1800757	0.951955786	99.78955189	0.450753967	-1.118966889	0.59922473
*Adam22*	a disintegrin and metallopeptidase domain 22	88.31886637	0.935023936	180.7780221	0.586164894	-2.046878878	0.359894666
*Adam8*	a disintegrin and metallopeptidase domain 8	86.51484226	0.899121169	92.0647805	0.35224885	-1.064150128	0.27105888
*Adamts13*	a disintegrin-like and metallopeptidase with thrombospondin type 1, 13	85.83956723	0.885539162	88.47392725	0.309067767	-1.030689344	0.478844207
*Adamts9*	a disintegrin-like and metallopeptidase with thrombospondin type 1, 9	83.27099934	0.833234446	189.3210225	0.518726499	-2.273552906	0.254563761
*Adamts18*	a disintegrin-like and metallopeptidase with thrombospondin type 1, 18	81.4196745	0.794981469	168.7064225	0.694045146	-2.072059653	0.162103461
*Adamts14*	a disintegrin-like and metallopeptidase with thrombospondin type 1, 14	80.27793265	0.771197631	82.77491987	0.244956077	-1.031104279	0.439155995
*Adamts19*	a disintegrin-like and metallopeptidase with thrombospondin type 1, 19	69.27461445	0.539213852	109.3590982	0.579553669	-1.578631639	0.390223674
*Adamts4*	a disintegrin-like and metallopeptidase with thrombospondin type 1, 4	68.27128822	0.518287473	74.72450982	0.165658834	-1.094523214	0.478842805
*Adam21*	a disintegrin and metallopeptidase domain 21	60.66601728	0.36550739	64.64919733	0.088605328	-1.065657517	0.999197582
*Adamts20*	a disintegrin-like and metallopeptidase with thrombospondin type 1, 20	58.83741845	0.331040492	97.20251711	0.417043299	-1.652052719	0.478849238
*Adam12*	a disintegrin and metallopeptidase domain 12	55.75657162	0.275742738	151.6194647	0.872877811	-2.719311111	0.196227734
*Adamts5*	a disintegrin-like and metallopeptidase with thrombospondin type 1, 5	55.55758605	0.272304468	141.9022559	0.987828457	-2.554147254	0.09184658
*Adamts3*	a disintegrin-like and metallopeptidase with thrombospondin type 1, 3	51.72989842	0.209742874	170.6776655	0.675392791	-3.299400748	0.478860496
*Adamts15*	a disintegrin-like and metallopeptidase with thrombospondin type 1, 15	51.52479453	0.206595412	79.74566033	0.213438929	-1.547714281	0.219403185
*Adamtsl3*	ADAMTS-like 3	47.33432389	0.147349513	66.40359283	0.10011701	-1.402863448	0.67686266
*Adam23*	a disintegrin and metallopeptidase domain 23	43.57759051	0.103048326	117.4744258	0.69092512	-2.695753125	0.127323609
*Adamts6*	a disintegrin-like and metallopeptidase with thrombospondin type 1, 6	40.47668384	0.073119037	142.2520509	0.983536216	-3.514419598	0.153396483

The ligands specific for ERBB2 are unknown, but ERBB2 can dimerize with EGFR. The heterodimers, similar to the EGFR homodimers, can be activated by amphiregulin (AREG), heparin-binding EGF-like growth factor (HB-EGF) and transforming growth factor α (TGFα) [58]. Activation of the EGFR signaling is essential for embryonic eyelid closure [59]. Based on the relative abundance of receptor gene expression, the EGFR/EGFR and EGFR/ERBB2 dimers are likely to form in the developing eyelid epithelium. Specifically, the EGFR/ERBB2 may be the dominant form in LE, whereas the EGFR/EGFR is likely to be the predominant form in IE cells.

ADAMS10 is important for the development of blood vessels and central nervous system, as well as in pathological conditions such as inflammation and cancer [60]. Recently, it was shown that ADAMS10 may be the sheddase of notch receptors, involved in the release of the extracellular domain and mediating skin development; however, its role in eyelid development has not been established. On the other hand, the *Adams17* knockout mice exhibit the open eye phenotype [61]. ADAMS17 is the major sheddase of TGFα, amphiregulin, HB-EGF and epiregulin, and is essential for activation of EGFR during development [62], [63]. Of the *Lgr/Gpcr* families, only the *Lgr4 (−/−)* mice have defective keratinocyte motility and produce the EOB phenotype. The *Lgr4*, also known as *Gpr48*, was known to play a role in HB-EGF-induced EGFR activation [64], [65]. The expression of *Adams17* and *Lgr4* was both relatively abundant in the IE cells (Tables 4 and 5).

The most surprising observation made by the RNA array was that expression of EGFR ligands was scarce in the LE and IE cells (Table 3). This was in clear contrast to previous findings made by *in situ* hybridization and immunohistochemistry, which showed that expression of TGFα and HB-EGF was up-regulated in a group cells located at the tip of the developing eyelid [6], [38], [66]. The discrepancy could be explained if induction of ligand is a temporal-spatial event, taking place in a small number of cells and in a narrow window during embryogenesis. Hence, either ligand up-regulation was insignificant at E15.5, or the expression signals were masked or under-represented in the collectives of the LCM captured cells, exemplifying the limitations of this approach.

Taken together, the gene expression data confirm that many genetically identified “eyelid closure” factors, such as FGFR, EGFR, ADAMS17 and LGR4, are also relatively abundant in the LE and/or IE cells, but some highly expressed genes, including *Fgf9* and *Adam10*, are not known to be involved in eyelid closure. In comparison to the IE cells, the LE cells have higher expression of *Fgfr2* and *Erbb2*, which may contribute to differential signaling responses of these cells.

### Expression of genes involved in the TGFβ signaling

The TGFβ superfamily consists of more than 30 structurally related ligands. They belong to the Bone Morphogenetic Proteins (BMPs), TGFβs and Activin/Inhibin subfamilies [67]. These ligands act selectively on seven type I and five type II receptors, resulting in receptor dimerization and activation. The receptors in turn activate two sets of so called R-SMAD. SMAD 1, 5, and 8 are substrates of Type I receptors for BMPs, whereas SMAD2 and 3 are substrates for Type I receptors for TGFβs and Activins. Once activated, R-SMADs assemble with SMAD4, also known as co-SMAD, and the heterodimer translocates into the nucleus to regulate responsive gene expression.

In LE and IE cells, the *Acvr2a* was the significantly expressed receptor gene, while *Smad2* was the abundantly expressed gene for intracellular transmitter. In addition, expression of *Bmp7* was relatively abundant in LE, and *Growth differentiation factor 10* (*Gdf10*) was abundant in IE cells (Table 6). Furthermore, the IE cells had a slightly higher expression of *inhibin beta-B*, but much higher *Bmp5*, *Bmpr1a* and *Acvr1*.

**Table 6:** Expression of genes in the TGFβ pathways

		LE		IE		LE/IE	
symbol	name	ave.int	p-val	ave.int	p-val	fold	p.val
**The TGFβ family**							
***Ligands***							
*Bmp7*	bone morphogenetic protein 7	273.8032153	0.067190002	164.4743744	0.782003153	1.664716564	0.108606617
*Inhbb*	inhibin beta-B	189.8249605	0.267867653	229.6743744	0.349920499	-1.209927155	0.015593156
*Bmp4*	bone morphogenetic protein 4	187.94911	0.276418245	187.8661392	0.590049741	1.000441648	0.143749699
*Gdf10*	growth differentiation factor 10	183.9074068	0.295778825	359.5450161	0.069068988	-1.95503282	0.661905204
*Bmp2*	bone morphogenetic protein 2	182.4613117	0.303028915	135.916909	0.921038359	1.342447478	0.212844348
*Bmp1*	bone morphogenetic protein 1	177.4112267	0.329755901	223.090068	0.380257649	-1.257474356	0.254597392
*Tgfb2*	transforming growth factor, beta 2	172.6298162	0.35719415	300.8077524	0.142523738	-1.742501724	0.329864054
*Bmp8a*	bone morphogenetic protein 8a	172.1103393	0.360306504	164.7585892	0.779391571	1.044621346	0.089033623
*Gdf11*	growth differentiation factor 11	159.4675951	0.44467919	175.0468033	0.689538262	-1.097695135	0.201245908
*Bmp3*	bone morphogenetic protein 3	148.1166656	0.536120106	202.4442107	0.492685372	-1.366788875	0.477486266
*Gdf7*	growth differentiation factor 7	147.8721307	0.538269393	153.7647042	0.8854851	-1.039849115	0.156938123
*Inhba*	inhibin beta-A	130.5738373	0.711324184	197.0395548	0.52691848	-1.509027833	0.712026446
*Inha*	inhibin alpha	130.1272773	0.716364388	204.5980265	0.479628814	-1.572291611	0.237387534
*Bmp6*	bone morphogenetic protein 6	125.274918	0.773059365	146.8885336	0.957046207	-1.172529473	0.286103958
*Nodal*	nodal	103.8101562	0.934420257	85.88759992	0.315528911	1.208674551	0.247190059
*Nog*	noggin	100.0350956	0.876554259	83.19324041	0.286197111	1.202442592	0.195250499
*Tgfb1*	transforming growth factor, beta 1	82.15218608	0.586613596	155.2142228	0.87090424	-1.889349879	0.210538535
*Bmp8b*	bone morphogenetic protein 8b	81.76056963	0.580144573	102.4426736	0.511615715	-1.252959391	0.578881901
*Inhbc*	inhibin beta-C	74.08385854	0.454687654	82.33694939	0.277085231	-1.111402011	0.542313176
*Inhbe*	inhibin beta E	69.57742791	0.383541072	74.89766277	0.202836798	-1.076464955	0.479105423
*Tgfb3*	transforming growth factor, beta 3	66.35517701	0.334591515	165.0505869	0.776715748	-2.487380704	0.254565854
*Gdf9*	growth differentiation factor 9	64.46053434	0.306763225	71.94859953	0.176159291	-1.116165112	0.164059633
*Gdf6*	growth differentiation factor 6	56.98847182	0.205865955	140.1852498	0.969536519	-2.459887856	0.274524202
*Gdf3*	growth differentiation factor 3	55.99339008	0.193684893	108.15508	0.582739207	-1.931568705	0.511633304
*Gdf5*	growth differentiation factor 5	51.66075472	0.14467988	60.11493281	0.087859418	-1.163647979	0.478845373
*Bmp5*	bone morphogenetic protein 5	51.55346944	0.143554136	314.8319068	0.11964688	-6.106900471	0.022920742
*Gdf2*	growth differentiation factor 2	50.43190792	0.132051407	52.21430519	0.047202918	-1.03534265	0.201766807
***receptor***							
*Acvr2a*	activin receptor IIA	**785.286694**	**0.006949311**	824.9994488	0.053983644	-1.050571027	0.535202305
*Bmpr2*	bone morphogenic protein receptor, type II	407.8628762	0.093450343	551.8506099	0.175491837	-1.353029761	0.371468892
*Bmpr1a*	bone morphogenetic protein receptor, type 1A	198.0829607	0.581647187	565.1672519	0.164924701	-2.853184594	0.028854135
*Crim1*	cysteine rich transmembrane BMP regulator 1	153.691022	0.876649887	204.0309462	0.951248863	-1.327539784	0.582802236
*Tgfbr3*	transforming growth factor, beta receptor III	143.0723153	0.965261747	200.257104	0.930110624	-1.399691503	0.323764976
*Bambi*	BMP and activin membrane-bound inhibitor, homolog	135.4573152	0.96669452	189.5920525	0.868484804	-1.399644251	1
*Tgfbr1*	transforming growth factor, beta receptor I	122.9008043	0.846582157	462.154534	0.27030705	-3.760386569	0.147558346
*Acvrl1*	activin A receptor, type II-like 1	116.9128312	0.786093483	136.2815587	0.525171941	-1.165668107	0.633311024
*Tgfbr2*	transforming growth factor, beta receptor II	112.9823508	0.745389803	163.7414235	0.70827237	-1.449265503	0.319010671
*Acvr1b*	activin A receptor, type 1B	101.4320918	0.622064638	110.4656142	0.350173625	-1.089059806	0.526331743
*Acvr1*	activin A receptor, type 1	100.3229914	0.609999096	247.9101709	0.82894344	-2.471120203	0.016173055
*Acvr2b*	activin receptor IIB	100.265421	0.609372034	111.8813641	0.359598279	-1.115851935	0.180539507
*Bambi-ps1*	BMP and activin membrane-bound inhibitor, pseudogene	80.5511445	0.393972944	80.94115829	0.168579167	-1.004841816	0.358188963
*Bmpr1b*	bone morphogenetic protein receptor, type 1B	76.22336452	0.347952441	116.7512861	0.392273989	-1.531699457	0.792545942
*Tgfbrap1*	transforming growth factor, beta receptor associated protein 1	73.74312689	0.32209407	141.4743747	0.560446165	-1.918475398	0.475558452
***intracellular***							
*Smad2*	MAD homolog 2 (Drosophila)	629.9527764	0.095677501	818.2805642	0.092838612	-1.298955406	0.195409967
*Smad4*	MAD homolog 4 (Drosophila)	354.4708292	0.364213634	447.8759579	0.391619063	-1.263505826	0.744635668
*Smad3*	MAD homolog 3 (Drosophila)	225.6921006	0.755336708	228.0715789	0.947457399	-1.010543029	0.158708217
*Smad5*	MAD homolog 5 (Drosophila)	219.8816039	0.781633462	368.1947529	0.555950137	-1.674513676	0.540729299
*Smad1*	MAD homolog 1 (Drosophila)	145.1756563	0.786661254	192.0409924	0.763436753	-1.322818145	0.39420827
*Smad6*	MAD homolog 6 (Drosophila)	110.418776	0.527526974	169.8452718	0.639157382	-1.538191944	0.835712394
*Smad7*	MAD homolog 7 (Drosophila)	96.23172938	0.41606715	127.125665	0.387073288	-1.321036895	0.180320764
*Crim1*	MAD homolog 9 (Drosophila)	59.54834067	0.147995136	84.36472138	0.154019199	-1.416743446	0.243731275

Previous genetic studies in mice have implicated TGF β signaling in eyelid closure. Huang *et. al*. carried out a methodical gene knockout study, in which each TGFβ cascade was specifically inactivated in ocular surface epithelium [13]. The results showed that BMP, but not TGFβ or activin, signaling was required for eyelid closure. The EOB phenotype was observed in mice lacking the type I BMP receptor genes, *Acvr1* and *Bmpr1a*, the R-Smad genes, *Smad 1* and *Smad5*, and the Co-Smad gene, *Smad 4*, but not in mice lacking the type II TGFβ receptor gene *Tgfbr2* and the activin/TGFβ-activated R-Smad genes, *Smad2* and *Smad3*. Conditional deletion of *Bmpr1a* in the ectoderm and overexpression of the inhibitory SMAD7 in keratinocytes also led to an EOB phenotype [68], [69]. Our data showed that although the LE and IE cells had type II BMP receptor expression, only the IE cells expressed abundantly the type I receptor BMPR1A. Hence, activation of the BMP pathway can be carried out mainly in the IE cells.

Of the ligands highly expressed in IE cells, BMP5 is required for chondrocytic activity during endochondral ossification, and its deficiency leads to a number of skeletal defects [70]. GDF10 is expressed in skeletal muscles but is dispensable for fetal development [71]. Recently, it was shown that GDF10, similar to TGFβ, can activate Smad2/3 and counteract the BMP signals [72]. Of the ligands highly expressed in LE cells, BMP7 is required for eye development, but is dispensable for eyelid closure [73]. The *inhibin βB* is required for embryonic eyelid closure; however, it may do so through a mechanism independent of SMAD [13], [20], [39]. These observations seem to support the idea that activation of the BMP pathways for eyelid closure is initiated by BMP4 produced by the the mesenchymal cells, but not ligands produced in the epithelial cells [13]. Collectively, the gene expression pattern has identified differential expression of *Bmpr1a*, *Inhbb* and *Bmp5* in the LE and IE cells, and suggests that the BMP pathways may be preferentially activated in the IE cells.

### Expression of genes involved in the canonical Wnt pathways

The canonical Wnt pathway is activated by binding of ligands to the Frizzled (FZD) receptors, seven-transmembrane proteins with 10 family members (FZD 1–10), and co-receptors, such as the low-density lipoprotein-related receptor protein-5 or -6 (LRP5/6) [74], [75]. The receptor signal is transduced by the Dishevelled (DVL), which are scaffold proteins that interact with diverse proteins, including kinases, phosphatases and adaptor proteins. Intracellular transduction of the Wnt signal is carried out by stabilization and cytosolic accumulation of the critical mediator, β-catenin. The β-catenin then translocates to the nucleus, binds with members of the T-cell factor (TCF)/lymphocyte enhancer factor (Lef) family of transcription factors to regulate target gene expression [76].

Wnt ligands are a family of secreted signaling proteins, consisting of 19 members in mammals [77]. Their activities are antagonized by the Secreted frizzled-related proteins (SFRPs) and the dickkopf homologs (DKKs). The SFRP is a family of secreted glycoproteins that may antagonize Wnt-mediated signaling by direct competitive interaction with Wnt ligands or by formation of non-signaling complexes with Frizzled proteins [78], [79]. The DKKs, also secreted cysteine-rich proteins, interact with and inhibit the Wnt co-receptor Lrp5/6 [80].

The array data showed that the *Fzd3* was the most abundant receptor and *Ctnnb1* and *Tcf4* were abundant intracellular transducers expressed in LE and IE cells. While *Sfrp2* was highly expressed in LE and IE cells, *Dkk2* and *Sfrp1* were abundantly expressed in the IE cells, and *Apcdd1* was abundant in the LE cells (Table 7). In addition, *Dkk2* was 4-fold more abundant in the IE cells, conversely, *Apcdd1* was 1.7-fold more abundant in the LE cells.

**Table 7:** Expression of genes in the Wnt pathways

		LE		IE		LE/IE	
symbol	name	ave.int	p-val	ave.int	p-val	fold	p.val
**Wnt**							
***Ligands***							
*Apcdd1*	adenomatosis polyposis coli down-regulated 1	483.5614722	0.053814884	277.7634161	0.800811376	**603.839414**	**0.011267485**
*Sfrp2*	secreted frizzled-related protein 2	**348.8687731**	**0.020922218**	**654.0519384**	**0.037660546**	9263.508027	0.174835606
*Sfrp4*	secreted frizzled-related protein 4	249.649771	0.112795852	316.9220374	0.273063678	914.2547726	0.40043203
*Sfrp1*	secreted frizzled-related protein 1	211.7568665	0.218783911	573.2261883	0.057477828	3684.148697	0.273488221
*Dkk1*	dickkopf homolog 1 (Xenopus laevis)	198.204605	0.277148921	180.9890829	0.736604814	269.0786174	0.391951411
*Dkk2*	dickkopf homolog 2 (Xenopus laevis)	177.2474096	0.398062954	**721.2937998**	**0.027019747**	**6559.921251**	0.012726284
*Wnt10a*	wingless related MMTV integration site 10a	176.4076412	0.403821253	184.4279311	0.717444422	245.8833545	0.478844672
*Wnt2b*	wingless related MMTV integration site 2b	160.0394529	0.532467804	182.5437831	0.727876583	219.871688	0.926769779
*Wnt5b*	wingless-related MMTV integration site 5B	142.8377647	0.70517094	175.1915846	0.770130122	185.4722475	0.479705404
*Wnt10b*	wingless related MMTV integration site 10b	142.5633067	0.708262603	116.9518638	0.798107856	178.6266175	0.478844678
*Wnt4*	wingless-related MMTV integration site 4	139.5806594	0.742557274	137.7167967	0.9727424	143.4919043	0.374613153
*Wnt6*	wingless-related MMTV integration site 6	135.1613805	0.795715493	160.3732491	0.863152443	156.5903932	0.078390545
*Wnt9b*	wingless-type MMTV integration site 9B	133.6151939	0.814972582	132.0830361	0.927644338	144.0370932	0.289503817
*Dkk3*	dickkopf homolog 3 (Xenopus laevis)	110.7032791	0.86283769	136.6161576	0.964067385	114.8293998	0.206504995
*Wnt7b*	wingless-related MMTV integration site 7B	109.9061131	0.850570159	103.0750617	0.669327096	164.2038904	0.135756387
*Wnt3a*	wingless-related MMTV integration site 3A	105.5491979	0.782655232	115.4051747	0.784199907	134.5947595	0.279318999
*Wnt9a*	wingless-type MMTV integration site 9A	103.910602	0.756793175	95.49126948	0.595623787	174.45677	0.259209416
*Wnt2*	wingless-related MMTV integration site 2	91.74384223	0.562599042	83.13988271	0.472430417	194.1954603	0.625010844
*Wnt8a*	wingless-related MMTV integration site 8A	87.51527434	0.495855788	84.11155152	0.48219671	181.4928897	0.68835004
*Wnt7a*	wingless-related MMTV integration site 7A	84.77441059	0.453355276	82.50036825	0.466000878	181.9189931	0.203647077
*Wnt11*	wingless-related MMTV integration site 11	84.42137039	0.447937944	103.3922602	0.672364507	125.5589334	0.30355669
*Dkkl1*	dickkopf-like 1	80.6658968	0.391301917	49.5193887	0.155264947	**519.5370763**	**0.007020038**
*Wnt5a*	wingless-related MMTV integration site 5A	77.85246206	0.350290283	212.9173909	0.57775569	134.7497973	0.113472971
*Wnt3*	wingless-related MMTV integration site 3	70.09772231	0.245673781	64.97157496	0.292388531	239.7416961	0.080993879
*Dkk4*	dickkopf homolog 4 (Xenopus laevis)	61.9542671	0.153300371	67.89828852	0.32063799	193.2218548	0.228653762
*Wnt16*	wingless-related MMTV integration site 16	**47.68335129**	**0.046137884**	41.67678582	0.097927067	486.9271889	0.129518192
***Receptors***							
*Fzd3*	frizzled homolog 3 (Drosophila)	**621.1053146**	**0.041393074**	**813.2745816**	**0.010731489**	57876.90198	0.478857045
*Fzd9*	frizzled homolog 9 (Drosophila)	421.0632821	0.122596071	392.1751139	0.199012898	2115.758764	0.87130258
*Dvl3*	dishevelled 3, dsh homolog (Drosophila)	413.3018893	0.128448795	402.3131688	0.183945914	2246.866375	0.154874206
*Lrp6*	low density lipoprotein receptor-related protein 6	361.5150568	0.177156701	431.6042255	0.146840648	2461.954923	0.583273548
*Lrpap1*	low density lipoprotein receptor-related protein AP-1	251.7744179	0.374371039	394.0853272	0.196076999	1284.058914	0.305691833
*Fzd6*	frizzled homolog 6 (Drosophila)	228.5769945	0.444226001	253.913148	0.59664302	383.105118	0.809625026
*Lrp1*	low density lipoprotein receptor-related protein 1	203.9297815	0.535517337	263.3174719	0.553577155	368.3854721	0.263717473
*Daam1*	dishevelled associated activator of morphogenesis 1	180.844634	0.640904694	283.032779	0.472862601	382.4464733	0.627127554
*Fzd7*	frizzled homolog 7 (Drosophila)	160.7760067	0.751649955	186.5780299	0.995186416	161.5536588	0.434764207
*Lrp4*	low density lipoprotein receptor-related protein 4	150.3594548	0.817266008	180.2743744	0.947588434	158.6759076	0.546664989
*Lrp12*	low density lipoprotein-related protein 12	149.1675011	0.825158313	308.3467702	0.386144171	386.3000201	0.392040335
*Fzd10*	frizzled homolog 10 (Drosophila)	143.5471885	0.863477711	146.15934	0.667091155	215.1837683	0.093092703
*Fzd5*	frizzled homolog 5 (Drosophila)	133.4039545	0.937417185	179.4493072	0.941246041	141.731225	0.268788395
*Lrp8*	low density lipoprotein receptor-related protein 8	129.9627772	0.963950394	173.179921	0.892234598	145.6598718	0.96540813
*Daam2*	dishevelled associated activator of morphogenesis 2	129.5253696	0.96737678	213.5127234	0.819485923	158.0568573	0.232134795
*Lrp5*	low density lipoprotein receptor-related protein 5	124.4582986	0.992036878	182.9363107	0.967875244	128.5891952	0.478848283
*Fzd1*	frizzled homolog 1 (Drosophila)	119.3852371	0.949735838	114.1795981	0.390301586	305.8794564	0.212940516
*Dvl2*	dishevelled 2, dsh homolog (Drosophila)	113.9854854	0.902859622	168.4318283	0.854195591	133.4419032	0.473187759
*Fzd2*	frizzled homolog 2 (Drosophila)	112.3533043	0.888313733	109.4746106	0.351250408	319.8666868	0.159410823
*Lrp3*	low density lipoprotein receptor-related protein 3	108.3015606	0.851451757	147.8738335	0.681889718	158.8256248	0.897879402
*Dvl1*	dishevelled, dsh homolog 1 (Drosophila)	107.1117074	0.84042385	145.3054246	0.659703892	**162.3633097**	**0.026996352**
*Lrp10*	low-density lipoprotein receptor-related protein 10	60.98575657	0.357857719	117.050989	0.414542002	147.1159889	0.47884479
*Fzd8*	frizzled homolog 8 (Drosophila)	55.12069055	0.294466513	85.06205341	0.170546176	323.2009761	0.478843194
*Frzb*	frizzled-related protein	52.66725106	0.26854277	167.7802652	0.848916887	62.04052699	0.356862886
*Lrp11*	low density lipoprotein receptor-related protein 11	44.59142388	0.18728742	85.39483827	0.172671979	258.2435451	0.202392746
*Lrp2*	low density lipoprotein receptor-related protein 2	40.27024362	0.147448705	95.17067762	0.239837412	167.9064298	0.254562864
*Fzd4*	frizzled homolog 4 (Drosophila)	31.47827903	0.07796015	128.7726731	0.515600438	61.05169179	0.187858824
*Lrp2bp*	Lrp2 binding protein	**26.76371598**	**0.04889444**	70.3777375	0.089199837	300.0422086	0.93675131
***Intracellular destruction complex***							
*Ctnnb1*	catenin (cadherin associated protein), beta 1	1048.175521	0.190192411	1125.036148	0.143218133	7318.734695	0.207958814
*Gsk3b*	glycogen synthase kinase 3 beta	676.0486341	0.434376095	825.1731378	0.278543966	2427.080521	0.920205733
*Apc*	adenomatosis polyposis coli	254.3465152	0.692184095	482.4879277	0.670699658	379.2256522	0.150092586
*Gsk3a*	glycogen synthase kinase 3 alpha	765.6427453	0.351520757	402.5362199	0.839207813	912.3398685	0.085845708
*Axin2*	axin2	223.7622736	0.582166117	202.2410415	0.521127278	429.3812343	0.249343351
*Axin1*	axin 1	164.4811794	0.357051665	192.2826903	0.48170679	341.4549736	0.332440932
*Apc2*	adenomatosis polyposis coli 2	98.85499999	0.124948223	94.26789929	0.114534278	863.1040594	0.101742905
***Nuclear Factors***							
*Tcf4*	transcription factor 4	986.4723963	0.251483716	1197.491493	0.248273344	3973.331888	0.889945086
*Tcf3*	transcription factor 3	190.5045881	0.48987916	179.8919114	0.57458275	331.552919	0.312585321
*Lef1*	lymphoid enhancer binding factor 1	234.9432668	0.648208795	173.6581248	0.553021659	424.8355608	0.207245367

Among the receptors highly expressed, FZD9 is required for bone morphogenesis and is a receptor for non-canonical Wnt that activates JNK, while DVL3 is required for cardiac outflow tract development [81]–[84]. Neither, however, is known to be involved in eyelid closure. Conversely, FZD3, involved in axonal outgrowth, and FZD6, required for hair patterning, can collaborate on eyelid closure. Knocking out both *Fzd3* and *Fzd6* causes “unfused eyelids” in 10% of the offsprings [85], [86]. Likewise, the *Lrp6(−/−)* mice display multiple defects, including open eyes [87]–[89]. Although the nuclear factor TCF4 has not been implicated in eyelid closure, TCF3, through interactions with β-catenin, is shown to be crucial for eyelid closure [36], [90].

Using the Wnt reporter mice, it was shown that Wnt activity is repressed overall in eyelid epithelium [36]. The repression is likely to be mediated by the expression of Wnt antagonists. On the one hand, the retinoic acid (RA)-Pitx2 pathway can induce the expression of Wnt antagonists in the periocular mesenchyme; while on the other hand, the BMP and FGFR2 pathways can activate the expression of Wnt antagonists in ocular surface epithelium [13], [91]. Our results showed that antagonists could indeed be produced in the LE and IE cells. Of the antagonists, SFRP4 is dispensable for fetal development; SFRP1 and SFRP2 have redundant functions in regulating embryonic patterning, and DKK2 is required for epithelial differentiation and eyelid closure [12], [92]–[94]. In addition, APCDD1 is a membrane-bound glycoprotein that can interact with WNT3A and LRP5 and inhibit Wnt signaling in a cell-autonomous manner [95]. Our data also suggested that the LE and IE cells might use distinct antagonists for Wnt inhibition.

In the Wnt reporter mice, it is also shown that the canonical Wnt pathway is activated in restricted areas of the developing eyelids [36]. Specifically, Wnt activity is induced in a small group of epithelial cells positioned at the transition zone between the palpebral conjunctiva and eyelid tip epidermis, so called mucocutaneous junction (MCJ) [96], [97]. Repression of Wnt in the MCJ cells results in failure of eyelid closure [36]. Hence, Wnt may establish distinct morphogenetic fields within the developing eyelids, so that activation takes place in MCJ, but repression occurs elsewhere. Isolation of the MCJ cells and characterizing their molecular signatures may help to understand the developmental roles of the temporal-spatial Wnt activity.

### Genes in the SHH, NOTCH and the PCP pathways

The Sonic Hedgehog ligands bind to the transmembrane receptor Patched (*Ptch*) to initiate pathway signaling [98]. In its inactive state, PTCH exerts an inhibitory effect on the signal transducer Smoothened (SMO), but upon ligand binding, the inhibition on SMO is released and downstream signaling occurs. This leads to the activation of the Gli transcription factors. We found that expression of *Ptch1*, *Smo* and *Gli2*, but not the ligand genes, was relatively abundant in IE and LE cells (Table 8). This is in agreement with the idea that activation of Shh pathway is dependent on *Ptch1* expression induced by the FGFR signaling in the eyelid epithelial cells, and the SHH expression induced by FGF10 in the periocular mesenchyme [6], [13]. Furthermore, many of the genes were expressed slightly but significantly higher in LE than in IE cells, suggesting that this pathway may be differentially activated in these cells.

**Table 8:** Expression of genes in the Shh pathways

		LE		IE		LE/IE	
symbol	name	ave.int	p-val	ave.int	p-val	fold	p.val
**The SHH pathways**							
***Ligands***							
*Ihh*	Indian hedgehog	147.1238905	0.343655971	163.4318248	0.257104942	-1.11084491	0.159752369
*Shh*	sonic hedgehog	114.5198084	0.921343416	90.82711316	0.707793588	1.260854875	0.057945482
*Dhh*	desert hedgehog	81.67346381	0.295695919	78.22022394	0.448188021	1.04414766	0.356208622
**Receptors**							
*Ptch1*	patched homolog 1	732.1286053	0.151756837	387.1397413	0.159961283	1.891122319	0.065360857
*Smo*	smoothened homolog (Drosophila)	232.128924	0.720827257	236.9900927	0.535847474	-1.020941676	0.043881503
*Ptch2*	patched homolog 2	177.2644254	0.916565611	129.2130979	0.724494389	**1.3718766**	**0.046898841**
*Ptchd2*	patched domain containing 2	62.24859935	0.381277507	81.98061543	0.279593571	-1.31698731	0.215221343
*Ptchd1*	patched domain containing 1	53.35715576	0.307765053	111.3612941	0.554778418	-2.087092023	0.350146107
**Nuclear factors**							
*Gli2*	GLI-Kruppel family member GLI2	323.275679	0.284969075	208.0211403	0.899972309	**1.554052047**	**0.014527635**
*Gli1*	GLI-Kruppel family member GLI1	254.0822594	0.875224653	194.1079797	0.351745286	1.308973798	0.095148044
*Gli3*	GLI-Kruppel family member GLI3	219.0576273	0.361664682	230.2743744	0.290554641	-1.05120455	0.513564801

The NOTCH cascade consists of NOTCH, its ligands, and intracellular signal transmitters. Mammals possess four different notch receptors, including NOTCH 1–4, which are membrane-tethered transcription factors. They are activated by the ligands of the Delta, Serrate, Lag-2 families. In LE and IE cells, expression of NOTCH ligands and receptors was overall low, but *Jag1* was 1.5-fold and *Notch 1* was 1.5-fold more abundant in the LE than in the IE cells (Table 9). The role of NOTCH in eyelid development however has been inconclusive. On the one hand, constitutive activation of NOTCH in periocular mesenchyme leads to abnormalities in cranial facial development and incomplete eyelid closure; on the other hand, genetic ablation of NOTCH signaling in ocular surface epithelium does not cause an EOB phenotype [12], [13], [99]–[101].

**Table 9:** Expresison of genes in the Notch pathways

		LE		IE		LE/IE	
symbol	name	ave.int	p-val	ave.int	p-val	fold	p.val
**The Notch pathway**							
**Ligands**							
*Jag1*	jagged 1	291.2881266	0.105374112	197.9402992	0.311677556	1.471595869	0.091068921
*Dlk2*	delta-like 2 homolog (Drosophila)	197.9473027	0.274112696	249.333394	0.127067789	-1.259594804	0.478878354
*Dlk1*	delta-like 1 homolog (Drosophila)	160.8057089	0.417447523	228.2743744	0.183766506	-1.419566358	0.713116482
*Jag2*	jagged 2	123.4294683	0.652072473	136.4169347	0.855040508	-1.105221764	0.044310212
*Cntn2*	contactin 2	90.82785012	0.973262036	135.9033179	0.861637408	-1.49627364	0.478848975
*Dll3*	delta-like 3 (Drosophila)	82.77031667	0.925984883	94.41873989	0.524248856	-1.140731891	0.145970717
*Dll1*	delta-like 1 (Drosophila)	58.175215	0.566824074	86.88336375	0.411054244	-1.493477312	0.478850038
*Dll4*	delta-like 4 (Drosophila)	50.38482269	0.44226217	74.30942025	0.241923917	-1.474837387	1
*Cntn1*	contactin 1	39.8413045	0.276648487	96.92132086	0.562892733	-2.432684423	0.478853114
*Cntn3*	contactin 3	29.72792312	0.137186651	79.6325516	0.309575696	-2.678712242	0.072194119
**Receptor**							
*Notch1*	Notch gene homolog 1 (Drosophila)	270.8341937	0.165136399	174.4555076	0.868921425	**1.552454247**	**0.049764805**
*Notch3*	Notch gene homolog 3 (Drosophila)	170.0357029	0.942106713	161.7710801	0.837205225	1.051088382	0.134639977
*Notch2*	Notch gene homolog 2 (Drosophila)	129.2094217	0.481897644	216.7538874	0.218496242	-1.677539335	0.202460017
*Notch4*	Notch gene homolog 4 (Drosophila)	126.7584274	0.44882783	132.3657333	0.23400882	-1.044236158	0.478848306

The non-canonical Wnt/planar cell polarity (PCP) pathway regulates cell orientation within the plane of a cell sheet and is involved in convergent extension during development [28], [102]. WNT5A, WNT5B, and WNT11 are the non-canonical WNT ligands, and FZD 3/6 and DVL are the receptors, which transmit signals through the core PCP proteins. The core is composed of cytoplasmic Prickled (PK), the transmembrane protein Van Gogh, the cadherin Starry/Flamingo (STAN/FMI), and the Ankyrin repeat protein Diego (DGO) [103], [104]. In addition, SEC24B is a cargo-binding component of the COPII vesicle coat [105]. The COPII vesicles are the primary pathway for active transport of secretary proteins from the ER to the Golgi. Though SEC24B is not a PCP core component, it selectively sorts VANGL2 into COPII vesicles thereby controlling PCP assembly and activity.

Expression of non-canonical Wnt ligands and core receptors was overall low in LE and IE cells with a few exceptions (Table 10). While expression of *Fzd3* and *Dvl3* was relatively abundant in LE and IE cells, expression of *naked cuticle 1 homolog* (*Nkd1*) was higher in LE, and expression of *Sec24b* was higher in IE cells. Genetic inactivation of many PCP genes, including *Fzd3/6*, *Dvl2*, *Vangl2*, *Scrb1*, *Ptk7* and *Celsr1*, as well as *Sec24b*, causes craniofacial developmental abnormalities, including open eyelids [27]–[33], [35], [106]. It is yet to be determined whether the eyelid defect is secondary to craniofacial abnormalities resulting from inactivation of the PCP pathways.

**Table 10:** Expression of genes in the PCP pathways

		LE		IE		LE/IE	
symbol	name	ave.int	p-val	ave.int	p-val	fold	p.val
***Ligands***							
*Wnt5b*	wingless-related MMTV integration site 5B	142.8377647	0.251818298	175.1915846	0.766909218	-1.226507465	0.479705404
*Wnt11*	wingless-related MMTV integration site 11	84.42137039	0.652672424	103.3922602	0.26497932	-1.224716677	0.30355669
*Wnt5a*	wingless-related MMTV integration site 5A	77.85246206	0.486496883	212.9173909	0.41319785	-2.734883204	0.113472971
***Receptors/co-receptors***							
*Fzd3*	frizzled homolog 3 (Drosophila)	621.1053146	0.147519425	813.2745816	0.09579658	-1.309398845	0.478857045
*Fzd6*	frizzled homolog 6 (Drosophila)	228.5769945	0.707784383	253.913148	0.843771153	-1.110842972	0.809625026
*Ptk7*	PTK7 protein tyrosine kinase 7	131.0165691	0.823649378	130.2743778	0.519053347	1.005697139	0.271910987
*Ror2*	receptor tyrosine kinase-like orphan receptor 2	117.9563668	0.737154026	147.612897	0.626132679	-1.251419496	0.347033389
*Ror1*	receptor tyrosine kinase-like orphan receptor 1	49.65911375	0.205977337	121.7021912	0.464979521	-2.450752379	0.254560774
***PCP core molecules***							
*Nkd1*	naked cuticle 1 homolog (Drosophila)	**497.1448806**	**0.019711524**	296.7290136	0.106660925	1.675417158	0.059347345
*Dvl3*	dishevelled 3, dsh homolog (Drosophila)	**413.3018893**	**0.042567398**	**402.3131688**	**0.02480151**	1.027313847	0.154874206
*Nkd2*	naked cuticle 2 homolog (Drosophila)	190.6089541	0.450452887	228.3393772	0.284509583	-1.19794675	0.254709977
*Celsr1*	cadherin, EGF LAG seven-pass G-type receptor 1	143.6045951	0.772719535	135.8743755	0.995259555	1.056892402	0.06910682
*Scrib*	scribbled homolog (Drosophila)	141.4973543	0.791394792	147.7381109	0.866894575	-1.044105111	0.121047968
*Vangl1*	vang-like 1 (van gogh, Drosophila)	131.9203286	0.88139334	183.465837	0.537481643	-1.390732111	0.345788803
*Dvl2*	dishevelled 2, dsh homolog (Drosophila)	113.9854854	0.927335626	168.4318283	0.660389528	-1.477660316	0.473187759
*Celsr2*	cadherin, EGF LAG seven-pass G-type receptor 2	112.3460222	0.908420016	106.673064	0.611755773	1.053180794	0.021674089
*Dvl1*	dishevelled, dsh homolog 1 (Drosophila)	107.1117074	0.846548722	145.3054246	0.894048687	**-1.35657836**	**0.026996352**
*Ankrd6*	ankyrin repeat domain 6	90.63533762	0.639562999	97.05578363	0.481777055	-1.07083822	0.479084068
*Prickle3*	prickle homolog 3 (Drosophila)	84.83043606	0.563803071	79.27375338	0.261432352	1.07009486	0.177745976
*Prickle4*	prickle homolog 4 (Drosophila)	80.8529364	0.511691571	106.7370389	0.612627926	-1.320138064	0.286671885
*Celsr3*	cadherin, EGF LAG seven-pass G-type receptor 3	79.55497285	0.494713944	86.50478832	0.346172204	-1.087358656	0.355310588
*Vangl2*	vang-like 2 (van gogh, Drosophila)	75.68020564	0.444286165	94.43627767	0.447152767	-1.247833259	0.215185176
*Prickle2*	prickle homolog 2 (Drosophila)	65.94364237	0.321422465	109.7444823	0.653633857	-1.664216266	1
*Prickle1*	prickle homolog 1 (Drosophila)	55.46965977	0.201923392	73.22103016	0.197860129	-1.320019457	0.912533584
***COPII vesicle***							
*Sec24b*	Sec24 related gene family, member B	365.8694535	0.189701062	**499.6743376**	**0.03653965**	-1.365717561	0.217269914
*Sec24c*	Sec24 related gene family, member C	334.7646094	0.254211948	299.8711063	0.603744187	1.116361671	0.170013082
*Sec24a*	Sec24 related gene family, member A	259.536154	0.51602466	329.3725376	0.968581141	-1.269081523	0.67083081
*Sec23ip*	Sec23 interacting protein	138.61087	0.575686004	308.2026826	0.704727443	-2.223510194	0.218337641
*Sec23b*	SEC23B (S. cerevisiae)	128.5699587	0.481018944	308.1660899	0.704276804	-2.396874767	0.326855537
*Sec24d*	Sec24 related gene family, member D	126.0420706	0.457514143	272.5389393	0.313682332	-2.162285481	0.165560621
*Sec23a*	SEC23A (S. cerevisiae)	105.0683677	0.274034086	347.439549	0.815321433	-3.30679496	0.243266726

### Validation of differential gene expression by qRT-PCR

**Figure 1:**
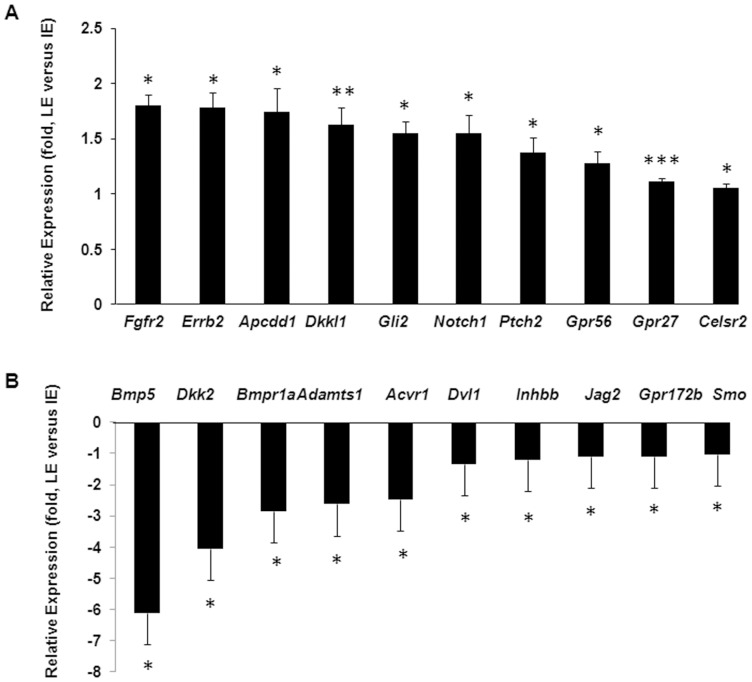
Summary of the microarray analyses. Genes differentially expressed in LE and IE cells. Statistical significant differential gene expression between LE and IE samples were summarized in (A) genes expressed more in LE than IE cells, and (B) genes expressed less in LE than IE cells. * p<0.05, **p<0.01 and ***p<0.001 are considered significant.

**Figure 2:**
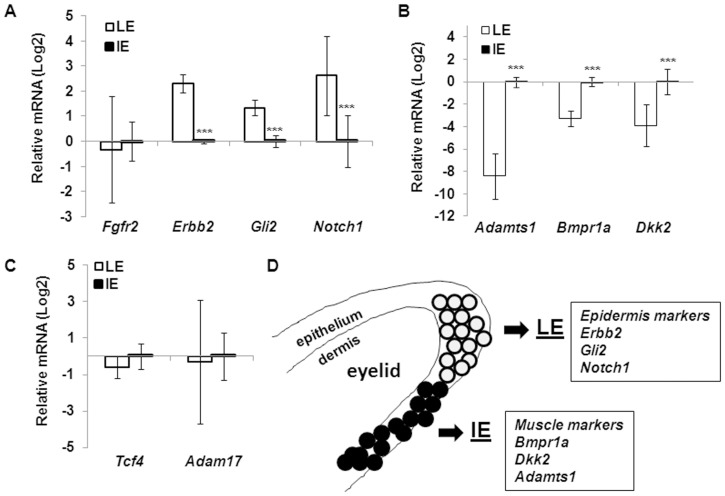
Differential gene expression in LE and IE cells. Total RNA isolated from LE and IE cells of fetuses at E15.5 was used for qRT-PCR for the expression of (A) *Fgfr2*, *Errb2*, *Gli2* and *Notch1*, (B) *Adamts1*, *Bmpr1a* and *Dkk2* and (C) *Tcf4* and *Adam17*. Relative expression was calculated based on that of *Gapdh* in each sample, and compared to the expression in IE cells, set as 1. The results are shown as mean ± SD from at least 3 samples and triplicate PCR of each sample. Statistic analyses were done by Student t-test, ***p<0.001 is considered significant. (D) Figure depicting the LE and IE cells in the developing eyelid and expresison of signaling factors.

Collectively, the microarray studies identified 20 genes of the morphogenetic signaling pathways were differentially expressed in the LE and IE cells (Fig. 1). To validate the results, we used qRT-PCR to examine 7 relatively abundant genes (Fig. 2A and 2B). Consistent with the array data, qRT-PCR showed that the LE cells had significantly more expression of *Erbb2*, *Gli2* and *Notch1*, but significantly less expression of *Adamts1*, *Bmpr1a* and *Dkk2* than the IE cells. Also consistent with the array data, qRT-PCR showed that the LE cells had a slight but insignificant decrease in expression of *Tcf4* and *Adam17* than the IE cells (Fig. 2C, Tables 4 and 7). Different from the array data, however, qRT-PCR detected no difference of *Fgfr2* expression in LE and IE cells (Fig. 2A). Hence, most gene expression pattern observed by cDNA array can be validated by qRT-PCR.

## Conclusions

The LE and IE cells have the same ontogenic origin, but different developmental fate. The fate divergence can be detected at E15.5, as the IE cells develop gene expression signatures towards the muscle lineage, while the LE cells express epidermal markers. The LE cells also undergo morphological changes and migrate at E15.5 to eventually form the closed eyelid. This morphogenetic event is thought to be dictated by specific activation of signaling pathways. Our results show that the LE and IE cells are overall quite similar in the compositions for the major “eyelid closure pathways”, but there are a few differences (Fig. 2D). The LE cells have a slight but significant increased expression of *Erbb2* of the EGF pathway, *Pach1*and *2* and *Gli2* of the Shh pathway, *Jag1* and *Notch 1* of the Notch pathway, and *Nkd1* of the PCP pathway, but the IE cells have higher expression of *Bmpr1a*, *Acvr1* and *Bmp5* of the BMP pathway. In addition, we find higher expression of *Apcdd1* in the LE cells, but higher expression of *Dkk2* in the IE cells of the Wnt pathway. Differential expression of signaling molecules in the eyelid epithelium may be one of the mechanisms for ectopic activation of morphogenetic pathways. The contributions of the eyelid mesenchyme should also be crucial and can be evaluated using the similar approach. Combination of LCM, cDNA array and pathway analyses can serve as a preliminary screening tool for identifying critical developmental genes for further expression and knockout studie.
