# Correction: Expression of Signaling Components in Embryonic Eyelid Epithelium

**DOI:** 10.1371/journal.pone.0106783

**Published:** 2014-08-26

**Authors:** The PLOS ONE Staff

The 7th column in Table 7, labelled “Fold,” is incorrect. The authors have provided a corrected version of Table 7 below.

**Table 7:** Expression of genes in the Wnt pathways.

		LE		IE		LE/IE	
symbol	name	ave.int	p-value	ave.int	p-value	fold	p.value
**Wnt**							
*Ligands*							
*Apcdd1*	adenomatosis polyposis coli down-regulated 1	483.56	0.05381	277.8	0.8008	**1.741**	**0.011**
*Sfrp2*	secreted frizzled-related protein 2	**348.87**	**0.02092**	**654.1**	**0.0377**	-1.87	0.175
*Sfrp4*	secreted frizzled-related protein 4	249.65	0.1128	316.9	0.2731	-1.27	0.4
*Sfrp1*	secreted frizzled-related protein 1	211.76	0.21878	573.2	0.0575	-2.71	0.273
*Dkk1*	dickkopf homolog 1 (Xenopus laevis)	198.2	0.27715	181	0.7366	1.095	0.392
*Dkk2*	dickkopf homolog 2 (Xenopus laevis)	177.25	0.39806	**721.3**	**0.027**	**-4.07**	**0.013**
*Wnt10a*	wingless related MMTV integration site 10a	176.41	0.40382	184.4	0.7174	-1.05	0.479
*Wnt2b*	wingless related MMTV integration site 2b	160.04	0.53247	182.5	0.7279	-1.14	0.927
*Wnt5b*	wingless-related MMTV integration site 5B	142.84	0.70517	175.2	0.7701	-1.23	0.48
*Wnt10b*	wingless related MMTV integration site 10b	142.56	0.70826	117	0.7981	1.219	0.479
*Wnt4*	wingless-related MMTV integration site 4	139.58	0.74256	137.7	0.9727	1.014	0.375
*Wnt6*	wingless-related MMTV integration site 6	135.16	0.79572	160.4	0.8632	-1.19	0.078
*Wnt9b*	wingless-type MMTV integration site 9B	133.62	0.81497	132.1	0.9276	1.012	0.29
*Dkk3*	dickkopf homolog 3 (Xenopus laevis)	110.7	0.86284	136.6	0.9641	-1.23	0.207
*Wnt7b*	wingless-related MMTV integration site 7B	109.91	0.85057	103.1	0.6693	1.066	0.136
*Wnt3a*	wingless-related MMTV integration site 3A	105.55	0.78266	115.4	0.7842	-1.09	0.279
*Wnt9a*	wingless-type MMTV integration site 9A	103.91	0.75679	95.49	0.5956	1.088	0.259
*Wnt2*	wingless-related MMTV integration site 2	91.744	0.5626	83.14	0.4724	1.103	0.625
*Wnt8a*	wingless-related MMTV integration site 8A	87.515	0.49586	84.11	0.4822	1.04	0.688
*Wnt7a*	wingless-related MMTV integration site 7A	84.774	0.45336	82.5	0.466	1.028	0.204
*Wnt11*	wingless-related MMTV integration site 11	84.421	0.44794	103.4	0.6724	-1.22	0.304
*Dkkl1*	dickkopf-like 1	80.666	0.3913	49.52	0.1553	**1.629**	**0.007**
*Wnt5a*	wingless-related MMTV integration site 5A	77.852	0.35029	212.9	0.5778	-2.73	0.113
*Wnt3*	wingless-related MMTV integration site 3	70.098	0.24567	64.97	0.2924	1.079	0.081
*Dkk4*	dickkopf homolog 4 (Xenopus laevis)	61.954	0.1533	67.9	0.3206	-1.1	0.229
*Wnt16*	wingless-related MMTV integration site 16	**47.683**	**0.04614**	41.68	0.0979	1.144	0.13
*Receptors*							
*Fzd3*	frizzled homolog 3 (Drosophila)	**621.11**	**0.04139**	**813.3**	**0.0107**	-1.31	0.479
*Fzd9*	frizzled homolog 9 (Drosophila)	421.06	0.1226	392.2	0.199	1.074	0.871
*Dvl3*	dishevelled 3, dsh homolog (Drosophila)	413.3	0.12845	402.3	0.1839	1.027	0.155
*Lrp6*	low density lipoprotein receptor-related protein 6	361.52	0.17716	431.6	0.1468	-1.19	0.583
*Lrpap1*	low density lipoprotein receptor-related protein AP-1	251.77	0.37437	394.1	0.1961	-1.57	0.306
*Fzd6*	frizzled homolog 6 (Drosophila)	228.58	0.44423	253.9	0.5966	-1.11	0.81
*Lrp1*	low density lipoprotein receptor-related protein 1	203.93	0.53552	263.3	0.5536	-1.29	0.264
*Daam1*	dishevelled associated activator of morphogenesis 1	180.84	0.6409	283	0.4729	-1.57	0.627
*Fzd7*	frizzled homolog 7 (Drosophila)	160.78	0.75165	186.6	0.9952	-1.16	0.435
*Lrp4*	low density lipoprotein receptor-related protein 4	150.36	0.81727	180.3	0.9476	-1.2	0.547
*Lrp12*	low density lipoprotein-related protein 12	149.17	0.82516	308.3	0.3861	-2.07	0.392
*Fzd10*	frizzled homolog 10 (Drosophila)	143.55	0.86348	146.2	0.6671	-1.02	0.093
*Fzd5*	frizzled homolog 5 (Drosophila)	133.4	0.93742	179.4	0.9412	-1.35	0.269
*Lrp8*	low density lipoprotein receptor-related protein 8	129.96	0.96395	173.2	0.8922	-1.33	0.965
*Daam2*	dishevelled associated activator of morphogenesis 2	129.53	0.96738	213.5	0.8195	-1.65	0.232
*Lrp5*	low density lipoprotein receptor-related protein 5	124.46	0.99204	182.9	0.9679	-1.47	0.479
*Fzd1*	frizzled homolog 1 (Drosophila)	119.39	0.94974	114.2	0.3903	1.046	0.213
*Dvl2*	dishevelled 2, dsh homolog (Drosophila)	113.99	0.90286	168.4	0.8542	-1.48	0.473
*Fzd2*	frizzled homolog 2 (Drosophila)	112.35	0.88831	109.5	0.3513	1.026	0.159
*Lrp3*	low density lipoprotein receptor-related protein 3	108.3	0.85145	147.9	0.6819	-1.37	0.898
*Dvl1*	dishevelled, dsh homolog 1 (Drosophila)	107.11	0.84042	145.3	0.6597	**-1.36**	**0.027**
*Lrp10*	low-density lipoprotein receptor-related protein 10	60.986	0.35786	117.1	0.4145	-1.92	0.479
*Fzd8*	frizzled homolog 8 (Drosophila)	55.121	0.29447	85.06	0.1705	-1.54	0.479
*Frzb*	frizzled-related protein	52.667	0.26854	167.8	0.8489	-3.19	0.357
*Lrp11*	low density lipoprotein receptor-related protein 11	44.591	0.18729	85.39	0.1727	-1.92	0.202
*Lrp2*	low density lipoprotein receptor-related protein 2	40.27	0.14745	95.17	0.2398	-2.36	0.255
*Fzd4*	frizzled homolog 4 (Drosophila)	31.478	0.07796	128.8	0.5156	-4.09	0.188
*Lrp2bp*	Lrp2 binding protein	**26.764**	**0.04889**	70.38	0.0892	-2.63	0.937
*Intracellular destruction complex*							
*Ctnnb1*	catenin (cadherin associated protein), beta 1	1048.2	0.19019	1125	0.1432	-1.07	0.208
*Gsk3b*	glycogen synthase kinase 3 beta	676.05	0.43438	825.2	0.2785	-1.22	0.92
*Apc*	adenomatosis polyposis coli	254.35	0.69218	482.5	0.6707	-1.9	0.15
*Gsk3a*	glycogen synthase kinase 3 alpha	765.64	0.35152	402.5	0.8392	1.902	0.086
*Axin2*	axin2	223.76	0.58217	202.2	0.5211	1.106	0.249
*Axin1*	axin 1	164.48	0.35705	192.3	0.4817	-1.17	0.332
*Apc2*	adenomatosis polyposis coli 2	98.855	0.12495	94.27	0.1145	1.049	0.102
*Nuclear Factors*							
*Tcf4*	transcription factor 4	986.47	0.25148	1197	0.2483	-1.21	0.89
*Tcf3*	transcription factor 3	190.5	0.48988	179.9	0.5746	1.059	0.313
*Lef1*	lymphoid enhancer binding factor 1	234.94	0.64821	173.7	0.553	1.353	0.207

## References

[1] Meng Q, Jin C, Chen Y, Chen J, Medvedovic M, et al (2014) Expression of Signaling Components in Embryonic Eyelid Epithelium. PLoS ONE 9(2): e87038 doi:10.1371/journal.pone.0087038 24498290 10.1371/journal.pone.0087038 PMC3911929

